# Genetic Diversity and Sequence Polymorphism of Two Genes Encoding *Theileria parva* Antigens Recognized by CD8^+^ T Cells among Vaccinated and Unvaccinated Cattle in Malawi

**DOI:** 10.3390/pathogens9050334

**Published:** 2020-04-30

**Authors:** Elisha Chatanga, Kyoko Hayashida, Walter Muleya, Kodai Kusakisako, Mohamed Abdallah Mohamed Moustafa, Bashir Salim, Ken Katakura, Chihiro Sugimoto, Nariaki Nonaka, Ryo Nakao

**Affiliations:** 1 Laboratory of Parasitology, Graduate School of Infectious Diseases, Faculty of Veterinary Medicine, Hokkaido University, Kita-18, Nishi-9, Sapporo, Hokkaido 060-0818, Japan; chatanga@vetmed.hokudai.ac.jp (E.C.); k.kusakisako@vetmed.hokudai.ac.jp (K.K.); m.abdallah@vetmed.hokudai.ac.jp (M.A.M.M.); kenkata@vetmed.hokudai.ac.jp (K.K.); nnonaka@vetmed.hokudai.ac.jp (N.N.); 2 Department of Veterinary Medicine, Lilongwe University of Agriculture and Natural Resources, P.O. Box 219 Lilongwe, Malawi; 3 Division of Collaboration and Education, Research Centre for Zoonosis Control, Hokkaido University, Kita-20, Nishi-10, Sapporo, Hokkaido 001-0020, Japan; kyouko-h@czc.hokudai.ac.jp (K.H.); sugimoto@czc.hokudai.ac.jp (C.S.); 4 Department of Biomedical Sciences, School of Veterinary Medicine, University of Zambia, P.O. Box 32379 Lusaka, Zambia; muleyawalter@gmail.com; 5 Department of Animal Medicine, Faculty of Veterinary Medicine, South Valley University, Qena City 83523, Egypt; 6 Department of Parasitology, Faculty of Veterinary Medicine, University of Khartoum, P.O. Box 32 Khartoum North, Sudan; bashirsalim@gmail.com

**Keywords:** Malawi, *Theileria parva*, genetic diversity, vaccine, Muguga cocktail

## Abstract

East Coast fever (ECF) is an acute fatal tick-borne disease of cattle caused by *Theileria parva*. It causes major losses in exotic and crossbreed cattle, but this could be prevented by a vaccine of *T. parva* if the vaccine is selected properly based on information from molecular epidemiology studies. The Muguga cocktail (MC) vaccine (Muguga, Kiambu 5 and Serengeti-transformed strains) has been used on exotic and crossbreed cattle. A total of 254 *T. parva* samples from vaccinated and unvaccinated cattle were used to understand the genetic diversity of *T. parva* in Malawi using partial sequences of the Tp1 and Tp2 genes encoding *T. parva* CD8^+^ antigens, known to be immunodominant and current candidate antigens for a subunit vaccine. Single nucleotide polymorphisms were observed at 14 positions (3.65%) in Tp1 and 156 positions (33.12%) in Tp2, plus short deletions in Tp1, resulting in 6 and 10 amino acid variants in the Tp1 and Tp2 genes, respectively. Most sequences were either identical or similar to *T. parva* Muguga and Kiambu 5 strains. This may suggest the possible expansion of vaccine components into unvaccinated cattle, or that a very similar genotype already existed in Malawi. This study provides information that support the use of MC to control ECF in Malawi.

## 1. Introduction

The apicomplexan tick-borne haemoparasite *Theileria parva* is the causative agent of bovine theileriosis (East Coast fever: ECF). The parasite, which is transmitted by the brown ear tick (*Rhipicephalus appendiculatus*), infects the T and B lymphocytes of cattle [1]. *T. parva* exhibits a complex life cycle [2] involving two stages, i.e., the schizont stage, which is responsible for the pathology of the disease and the blood stage, which is infective to the vector tick [1,3]. In lymphocytes, the parasite develops into a schizont through the process of schizogony in which the infected cells divide into a clonally proliferating lymphoblast [1]. During the pathogenesis of the disease, the parasite changes numerous signaling pathways of the infected host lymphocytes, resulting in the transformation of the infected host cells into a cancer-like phenomenon and eventually the infected host cells disseminate throughout the body of the animal, resulting in the enlargement of superficial lymph nodes, pulmonary edema and an eventual fatal fever [1,3,4,5,6]. ECF remains a major acute and usually lethal disease of cattle present in 13 countries in eastern, central and southern Africa including the Comoro Islands [4,7,8] and now South Sudan [9] in the north and Cameroon in the west [10]. 

Exotic and crossbreed cattle (*Bos taurus*) are more susceptible to ECF than the indigenous zebu cattle (*Bos indicus*) [11]. However, indigenous calves younger than 6 months of age are also highly susceptible [10]. Major losses due to ECF have also been reported even in adult indigenous zebu cattle, which are normally resistant to clinical disease [12,13,14]. This occurs when endemic stability, which is an epidemiological state in which clinical disease is rare in a population with high levels of infection, is disturbed [14,15,16]. ECF is responsible for nearly half of all calf deaths in pastoral herds in endemic countries [17]. It is estimated that 1 million cattle succumb to ECF annually with a projected annual loss of US$300 million and some 25 million cattle remain at risk. Twenty-one million of these are owned by small-holder farmers [17]. In Malawi, it was reported that calf mortality in indigenous Malawi zebu cattle due to ECF accounted for almost 66% of the annual calf crop [18]. This disease is therefore a major limiting factor to the expansion of the livestock sub-sector in the affected countries [19]. This highlights the need to prioritize the management and control of ECF in Malawi to achieve the vision of the country being self-sufficient in safe, locally produced livestock and livestock products [20].

Despite tick-borne diseases (TBDs) being widespread in all the three geographical regions (northern, central and southern) of Malawi, very few studies have been done. Among these studies or reports [13,21], ECF was a single major cause of cattle mortality in the central and the northern regions of Malawi. However, sporadic cases attributed to the illegal movement of animals from endemic areas have also been reported in the southern region [13]. The control of TBDs in Malawi has mainly focused on vector control by an acaricide application administered through community cattle dips, livestock movement controls, chemotherapy [13,21] and immunisation with the Muguga cocktail (MC) vaccine containing Muguga, Kiambu 5 and Serengeti-transformed strains in the central and the northern regions predominantly in exotic and crossbreed cattle [13,22]. The vaccine is sourced from the African Union Centre of Excellence for Ticks and Tick-borne Diseases (AU-CTTBD), Lilongwe, Malawi [22]. Currently, dipping occurs predominantly in commercial farms keeping exotic and crossbreed cattle, where it is done weekly or fortnightly in the rainy season and the dry season, respectively, and not in the local Malawi zebu [20]. Treatment with buparvaquone is done in both exotic and local Malawi zebu cattle when clinical diseases occur [20]. 

In order to determine the right combination of parasites to be used in live cocktail vaccines, it requires information generated from molecular epidemiological studies in a particular geographical location [1,23]. In the follow-up studies to evaluate the impact of MC in the southern province of Zambia after the introduction of the MC vaccine from 1985 until 1992, a possible clonal expansion of one of the MC components was observed [1]. Due to this observation, it was recommended that the MC should not be used in the province. Instead, a local strain approach using the *T. parva* Chitongo strain isolated from the same province was introduced to immunize cattle in a southern province of Zambia [22]. The transmission of the Kiambu 5 vaccine strain from vaccinated to unvaccinated cattle through ticks of the animals that co-graze for more than 1 year has been demonstrated [12]. These observations emphasize the need to conduct follow-up studies where the “infection and treatment” method with a cocktail vaccine is being used. Such studies provide indisputable epidemiological data based on which effective control measures can be conceived on whether to use a local strain approach, in which a broadly protective local stock of *T. parva* is used, or a cocktail approach, where a combination of stocks is used to provide broad immunity over a wider geographical area of the ECF endemic region [1]. 

Genes that encode for the *T. parva* Tp1 and Tp2 antigens havesuppl been demonstrated to be highly dominant targets of the CD8^+^ T cell response in cattle and provide protective immunity for *T. parva* in only a subset of immunized cattle with specific major histocompatibility complex (MHC) class I gene products of the A18 and A10 haplotypes (about 30%), respectively [24]. There is a profound immunodominant response to Tp1 and Tp2 in cattle of these MHC class I haplotypes to *T. parva*, and this is a major determinant of the parasite strain specificity of the response and hence the immune protection [25]. Thus, the Tp1 and Tp2 genes are good genetic markers to evaluate the impact of the vaccination. These can also be used to characterize the strains of *T. parva* and to study the nature and selection forces affecting the diversity in these antigens that stimulate T cell responses in cattle [25,26,27,28]. 

This study intends to evaluate the endemicity and genetic diversity of *T. parva* in Malawi through sequence and amino acid variant analysis of the *T. parva* CD8^+^ cytotoxic T cell antigen’s Tp1 and Tp2 genes. The study also intends to assess the similarity of the field parasite population to the MC vaccine components. This information will cover the knowledge gap that has existed since Malawi started using the MC to control ECF in 1984 and to help monitor the impact of this control method using molecular techniques. 

## 2. Results 

### 2.1. Theileria parva Screening

Nested polymerase chain reactions (PCRs) for the p104 gene used to screen the cattle blood samples (n = 446) showed a *T. parva* positive detection rate of 54.5% (243/446) from the three districts, as shown in Table 1. There was a statistically higher significance in the infection rate in the vaccinated animals (73.3%, *X^2^* = 4.34, *df* = 1, *p* = 0. 037227), whereas 46.9% of the unvaccinated cattle were positive for *T. parva* within Lilongwe, where the MC vaccine was used. There was also a statistically positive association between the age of the animals and the *T. parva* infection rate (*X^2^* = 15.9271, *df* = 2, *p* = 0.000348). There was a higher positive detection rate in adult cattle than calves, which may be due to the higher exposure to vector ticks in adult animals as compared with calves. There was also a statistically positive correlation between the sampling site and the *T. parva* positive detection rate (*X^2^* =19.7667, *df* = 2, *p* = 0.000051). However, with regard to sex, there was statistically no significant correlation with the *T. parva* positive detection rate (*X^2^* = 0.3698, *df* = 1, *p* = 0.543121). Similarly, there was statistically no significant correlation between the breed of cattle with the *T. parva* positive detection rate (*X^2^* = 0.7831, *df* = 1, *p* = 0.376194).

#### 2.1.1. Sequencing Analysis

**Figure 1:**
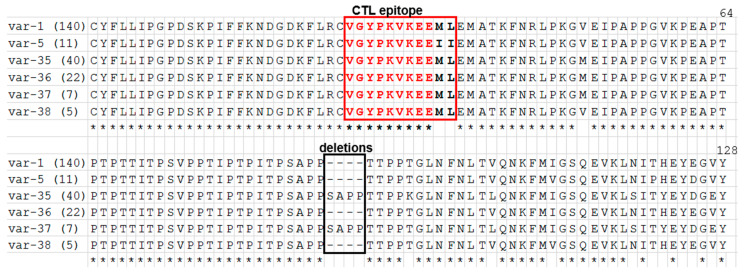
Multiple amino acid sequence alignment of 6 Tp1 amino acid variants in 223 *T. parva* samples obtained from cattle in Malawi. Var-1 to var-38 are names of the Tp1 antigen variants. Amino acid is represented by a single letter code. The naming of the antigen variants follows the nomenclature initiated by Pelle et al. [27]. Tp1 variants (var-1 and var-5) were reported previously by Pelle et al. [27]. The numbers in parenthesis after the variant name shows the number of *T. parva* isolates represented by each variant. The *T. parva* CD8^+^ T cell target epitope mapped in Tp1 is bolded and red boxed. The conserved amino acid residues in the epitope region are coloured in red. Conserved amino acid residues are denoted by (*) below the alignment, while dashes (–) denote the deletion region. Tp1 antigen variant var-1 is found in the three MC vaccine stocks (Muguga, Kiambu5 and Serengeti-transformed). Corresponding gene alleles are presented in Figure S1.

A total of 243 *T. parva* positive samples (22 vaccinated and 221 unvaccinated) from three districts in central Malawi (Table 1) were used to study the sequence polymorphisms in the partial sequences of the Tp1 and Tp2 genes. Single nucleotide polymorphisms (SNPs) were observed at 14/384 positions (3.65%) in Tp1 and 156/471 positions (33.12%) in Tp2, plus a deletion of 12 nucleotides (TCT GCA CCT CCT) translating into four amino acid residues SAPP in Tp1 (Figure 1) that gave rise to 6 and 10 amino acid variants in Tp1 and Tp2, respectively. The naming of the nucleotide alleles and the translated amino acid variants follows the nomenclature by previous studies that described nucleotide alleles 1 to 49 and amino acid variants 1 to 34 for Tp1 and nucleotide alleles 1 to 63 and amino acid variants 1 to 59 for Tp2 [23,27,28]. This study detected a total of 4 and 10 novel nucleotide alleles respectively for Tp1 and Tp2, leading to four and seven novel antigen variants for Tp1 and Tp2, respectively. These novel alleles or variants continued the numbering sequence from the ones indicated above. 

#### 2.1.2. Tp1 Gene 

We were able to obtain sequences coding for 128 amino acids residues from 223 *T. parva* positive samples in Tp1. We failed to obtain sequences from 20 samples. SNPs were observed at 14 positions (3.65%) and a short deletion of 12 nucleotides, resulting in 6 nucleotide alleles (Figure S1). The nucleotide polymorphism (π) in this region was 1.02%. The Tp1 allele number 1, present in the MC stocks, was observed in 139/223 (62.33%). There were six distinct amino acid variants, due to synonymous mutations at 11 positions (Figure 1). An analysis of the amino acid sequence of the single CD8^+^ T cell epitope in Tp1 showed that 212/223 (95.07%) had the sequence (VGYPKVKEE**ML**) present in the MC vaccine stocks. This was obtained in the samples from all the three sampled districts including both the vaccinated and the unvaccinated from Lilongwe. While 11/223 (4.93%) had the sequence (VGYPKVKEE**II**), this was observed in samples obtained from the Kasungu and Nkhotakota sampling sites with no MC vaccination history. Two of the six alleles identified in the Tp1 gene (allele IDs 1 and 5) were reported previously [29], while four (allele IDs 43 to allele 46) are reported here for the first time. Regarding the amino acid variants, two (variant-1 and variant-10) were reported previously [29], while four (variant-35 to variant-38) are reported here for the first time. 

#### 2.1.3. Tp2 Gene

**Figure 2:**
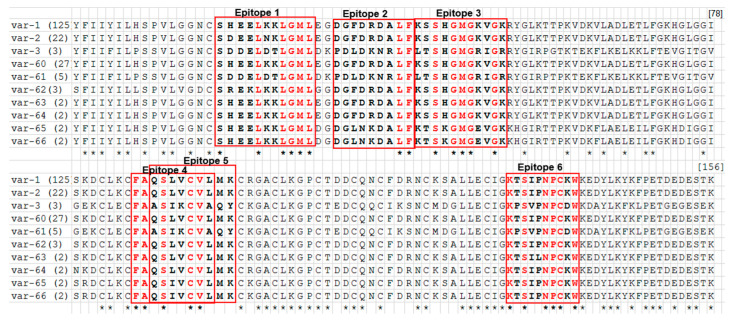
Multiple amino acid sequence alignment of the 10 Tp2 antigen variants in 190 *T. parva* samples obtained from cattle in Malawi. Variants var-1 to var-66 are names of the Tp2 antigen variants and amino acid is represented by a single-letter code. The naming of the variants follows the nomenclature initiated by Pelle et al. [29]. Tp2 antigen variants var-1, var-2 and var- 3 were reported previously by Pelle et al. [29]. The numbers in parenthesis after the variant name indicate the number of *T. parva* samples represented by each variant. Tp2 epitopes 1 to 6, that are the target of the bovine CD8^+^ T cells immune responses, are bolded and red boxed. The conserved amino acid residues in the epitope region are coloured in red. The star (*) below the alignment indicates the positions of the conserved amino acid residues. Tp2 Antigen variant var-1 is found in two MC vaccine stocks (Muguga and Serengeti-transformed), while Tp2 antigen variant var-2 is found in Kiambu5 vaccine stock. Corresponding gene alleles are presented in Figure S2.

We managed to obtain sequences coding for 156 amino acids residues from 190 *T*. *parva*-positive samples in Tp2. We failed to obtain sequences from 53 samples. SNPs were observed at 156 positions (33.12%), resulting in 13 nucleotide alleles (Figure S2). The nucleotide polymorphism (π) observed in this region was 3.05%. The Tp2 allele 1, present in two MC stocks (*T*. *parva* Muguga and Serengeti-transformed), was present in 118/190 (62.11%). Moreover, the Tp2 allele 2 present in the *T*. *parva* Kiambu 5 vaccine stock was found in 22/190 (11.58%). There were 10 distinct amino acid variants due to synonymous mutations at 76 positions (Figure 2). Further, an analysis of 190 Tp2 sequences showed that there were variations within the six epitopes mapped in this region. This resulted in four variants at epitope number 1 to three at epitope numbers 2, 3 and 6 and two at epitopes number 4 and 5 (Table 2). Among the 13 nucleotide alleles obtained in this study, 3 (allele IDs 1, 2 and 3) were reported previously [27], while the other 10 (allele IDs 64 to 73) are reported here for the first time. Likewise, from the 10 amino acid variants identified, 3 (variant IDs 1, 2 and 3) were reported previously [27], while the other 7 (variants-60 to variant-66) are reported here for the first time. 

#### 2.1.4. Phylogenetic Analysis of Tp1 and Tp2 Sequences from T. parva in Malawi 

**Figure 3:**
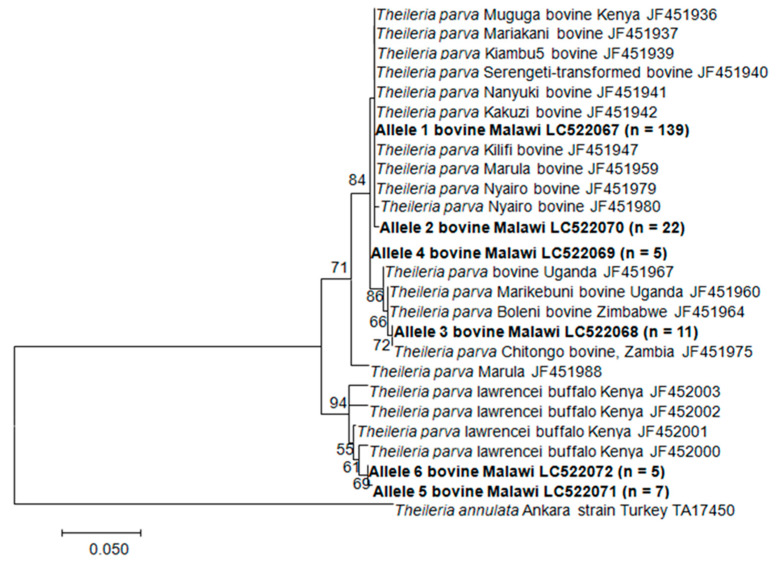
The maximum likelihood tree of the Tp1 gene sequences indicating phylogenetic relationships among the cattle-derived *T. parva* isolates. The partial sequences obtained in this study are in bold and the number in parenthesis is the number of sequences obtained in the allele. The sequence of the *T. annulata* Tp1 homologue (TA17450) was used to root the tree. Bootstrap values >50% are shown above the branches.

**Figure 4:**
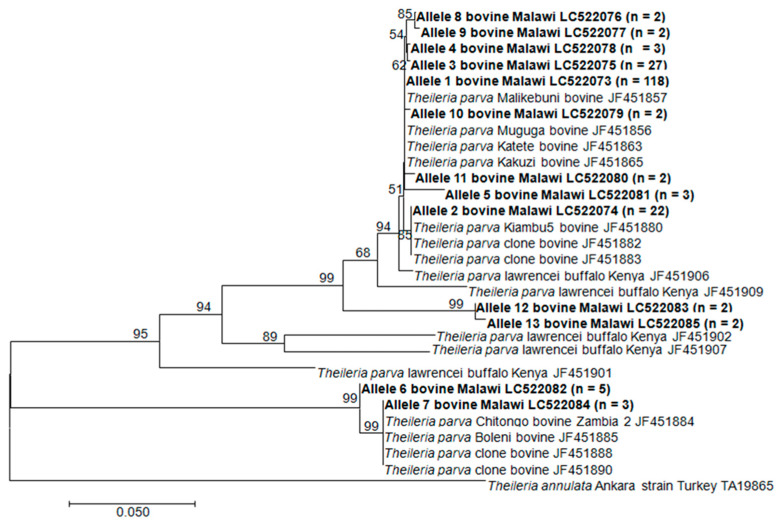
The maximum likelihood tree of the Tp2 gene sequences indicating phylogenetic relationships among the cattle-derived *T. parva* isolates. The partial sequences obtained in this study are in bold and the number in parenthesis is the number of sequences obtained in the allele. The sequence of the *T. annulata* Tp2 homologue (TA19865) was used to root the tree. Bootstrap values >50% are shown above branches.

To examine the relatedness of the Tp1 and Tp2 sequences generated in this study to those of the MC vaccine components, maximum-likelihood trees were created for both loci that were rooted with the orthologous nucleotide sequences of *T*. *annulata* (GenBank accession numbers TA17450 and TA19865 for the Tp1 and Tp2 genes, respectively). The Tp1 allele 1, which is found in the reference three MC vaccine components, had a majority of the sequences, 139 out of 223 (62.33%), including the sequences obtained from both the vaccinated and the unvaccinated animals. This allele also grouped together with the other eight closely related Tp1 alleles with a majority of the samples that originated from all three sampled districts (Figure 3; Figure 6). However, we observed that some sequences obtained from the Kasungu and Nkhotakota districts clustered together with those of the *T. parva* isolated from the buffaloes in Kenya. A phylogenetic analysis involving the 13 Tp2 alleles showed that the obtained sequences clustered into different clades with a majority of the sequences clustering together with the *T. parva* Muguga, Serengeti-transformed in the Tp2 allele 1, while *T. parva* Kiambu 5 was in the Tp2 allele 2 with the other 22 sequences. Two alleles clustered together with sequences isolated from the buffaloes in Kenya, as also observed in Tp1. The other two alleles clustered together with those of the Zambian Chitongo strain. The samples that did not cluster together with the reference Muguga isolate were from Kasungu and Nkhotakota. However, sequences identical to the one present in *T. parva* Kiambu 5 were only found in Lilongwe, where the *T. parva* MC vaccine was used, in both the vaccinated and the unvaccinated cattle (Figure 4). 

A genetic diversity analysis in Tp1 and Tp2, using the analysis of molecular variance (AMOVA), showed that in the Tp1 sequences, 50.5% of the variation happened within populations, while 43.03% of the variation was due to differences between populations (Table S1). However, for the Tp2 locus, 77.16% of the variation was within populations, while 20.06% was due to differences between populations (Table S2). 

**Figure 5:**
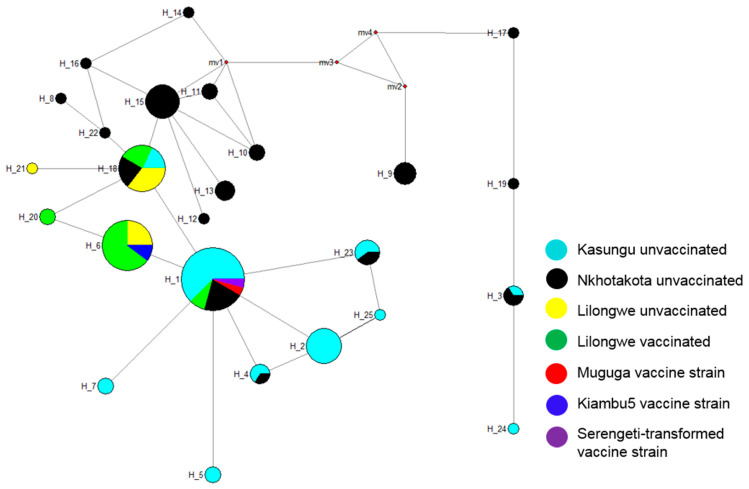
Median-joining network of concatenated (Tp1 + Tp2) nucleotide sequences using samples that have sequences in both loci. *T. parva* Muguga, Kiambu5 and Serengeti-transformed sequences were also included to compare their relatedness. The size of the circle is proportional to the haplotype frequencies. The origins of samples are colour coded.

The median-joining network (MJ) of the Tp1 and Tp2 *T*. *parva* haplotypes from Malawi was generated using concatenated Tp1 and Tp2 sequence alignments to generate a combined data matrix (Tp1 + Tp2), in order to optimize the network signal, is shown in Figure 5. The samples with data for a single locus were not included in the concatenated dataset. 

The haplotype H1 was represented by a majority of our sequences including the *T. parva* Muguga and Serengeti-transformed and samples from the vaccinated cattle in Lilongwe as well as the unvaccinated cattle from Kasungu and Nkhotakota. On the other hand, other haplotypes (haplotype IDs: H2, H4, H5, H6, H7, H18 and H23) were separated from H1 by mutations at two or three positions. We observed that five haplotypes (haplotype IDs: H3, H9, H17, H19 and H24) were disconnected from haplotype H1 by median vectors, which means these do not share a common ancestor with H1. However, the remaining 20 haplotypes were not disconnected by a median vector, which means that they share a common ancestor with H1. The haplotype H6 which includes the *T. parva* Kiambu 5 strain, clustered with sequences from both the vaccinated and the unvaccinated cattle in Lilongwe. This observation may imply that the vaccine stock has been transmitted to the unvaccinated cattle through ticks since both the vaccinated and unvaccinated graze together and the vector tick is present. Sequences from the vaccinated animals were also observed in haplotypes H1, H18 and H20, which were separated from haplotype H6 by mutations at the 2 to 3 positions. 

## 3. Discussion 

The overall *T. parva* positive rate of 54.5% (243/446) observed in this study using the nested p104 PCR assay is comparable to that reported from the neighboring countries such as Zambia and Tanzania, where overall positive rates were 54.9% and 62%, respectively [19,30]. Since the sampled animals were apparently healthy, this may suggest that the endemic stability against ECF in these southern African countries is well established in the local zebu cattle. Furthermore, the absence of clinical disease despite the presence of infection in the vaccinated exotic breed cattle may suggest that the MC vaccine worked as it was designed to prevent the occurrence of clinical disease but not infection [12,13]. 

A Tp1 and Tp2 genes sequence analysis has been used to investigate the genetic diversity of *T. parva* in Kenya [29], South Sudan [23], Tanzania [30,31], DR Congo and Burundi [32]. These studies have shown that the Tp1 gene is more conserved and has limited genetic diversity when compared with Tp2 which is highly polymorphic, which is in accordance with the results obtained in our study. It has also been reported that *T. parva* parasites isolated from buffaloes have a higher diversity when compared with cattle-derived parasites [29,31]. Furthermore, these studies have also shown that there is widespread polymorphism even within the epitope coding regions in both antigens. This observation is supported by the identification of new epitopes that have not been reported previously whenever a new study was conducted [23,31,32]. However, it has also shown that the majority of parasites in cattle share epitopes that are present in the *T. parva* Muguga strain, a component of the MC vaccine even in populations where the MC vaccine has not been deployed [23,29,31,32]. This is also supported by the results of the current study and warrants the deployment of the MC live vaccine in these ECF endemic countries as the most effective way to control the disease. This observation may also help to explain why the MC live sporozoite vaccine has been able to confer protection to immunized cattle over a wider geographical area. 

This polymorphism in the nucleotide sequences may also help to explain the difficulties in designing primers to amplify all samples that were positive by the *T. parva* species-specific PCR, as also observed in Kenya [29] and South Sudan [23]. Similarly, the nucleotide polymorphisms (μ) of 1.02% in Tp1 and 3.05% in Tp2 also support previous studies that there is higher polymorphism in Tp2 than in Tp1. This also translated into a higher level of polymorphism in amino acid residues of the Tp2 gene, as the alignment of the amino acid sequences showed that 77/156 (49.36%) residues were variable, while 51.64% were conserved. However, in Tp1, 116/128 (90.6%) of the residues were conserved, while 9.4% were variable. The failure to sequence a considerable number of the Tp2 gene may not provide a complete genetic diversity profile, which may result in underestimation as also reported by Salih et al. [23]. We detected more new epitope variants within the six Tp2 mapped epitopes in this study than those reported previously [23,29,31,32]. Thus, this finding may also provide evidence that there is genetic diversity in *T. parva* circulating in cattle in Malawi. 

The phylogenetic analysis revealed that some of the sequences obtained in Kasungu and Nkhotakota were closely related to the buffalo type reported in Kenya [29]. Sibeko et al. [33] reported that *T. parva lawrencei* (buffalo sp.) transmission to cattle can occur in the presence of vector ticks where contact with infected buffaloes or ticks is made susceptible in cattle. Our sampling sites in the Kasungu and Nkhotakota districts were less than 5 km from the Kasungu National Park and Nkhotakota Wildlife Reserve, respectively, where buffaloes are present as such so that we can speculate that some of these parasites were transmitted to cattle from buffaloes as the vector tick *R. appendiculatus* is present. However, *T. parva* has not been isolated from buffaloes in these areas. Thus, there is a need to conduct similar studies using samples collected from buffaloes in these areas. This may help to answer if *T. parva lawrencei* is present in Malawi. Information from such molecular epidemiological studies will provide indisputable data based on which effective control strategies can be conceived. 

In this study, we obtained Tp1 and Tp2 gene sequences that were identical to the MC vaccine strains in the unvaccinated cattle. This observation may imply that the vaccine components are expanding or that one of the local strains with similar genotypes is undergoing expansion. This may also be supported by a previous finding that the *T. parva* Kasowa strain isolated from the Karonga district in northern Malawi was antigenically similar to the *T. parva* Muguga strain [21]. However, the sequences identical to Kiambu 5 were only obtained in Lilongwe, where animals were vaccinated with MC 27 months before the sampling period, in both the vaccinated and the unvaccinated. This demonstrates the transmission of the Kiambu 5 vaccine strain to unvaccinated cattle [12,13]. It has also been documented that the *T. parva* Marikebuni vaccine strain can also be transmitted to unvaccinated cattle [34]. Despite the MC being made up of three strains, we could not obtain sequences related to the Serengeti-transformed. This was also observed in the previous reports, where researchers found only Muguga and Kiambu 5 but not Serengeti-transformed strains after the experimental inoculation of the MC against Friesian and Zebu cattle [12,13]. The transmission of a vaccine strain to unvaccinated cattle provides evidence that the MC live vaccine may also be contributing to the genetic diversity of *T. parva* in cattle in Malawi. However, the absence of pre-vaccination data makes it difficult to confirm this hypothesis. Nonetheless, the significance of this observation is that the MC may have resulted in the induction of the immune response in the unvaccinated animals that get infected with the vaccine strains, which is beneficial in the short-term but its long-term impact cannot be theoretically predicted. The absence of complications resulting from the presence of vaccine strains in cattle in Malawi may suggest that there is cross-protective immunity between the vaccine strains and the local *T. parva* strain circulating in cattle in Malawi, which is advantageous.

We have observed that the vector tick can transmit the vaccine stocks from the vaccinated to the unvaccinated cattle when they mix during grazing. Thus, vaccination of animals with the MC in non-endemic areas is not recommended as such a vaccination can result in disease outbreak in naïve animals, as in the case of the Comoros Islands, in which the introduction of the MC-vaccinated cattle from Tanzania preceded the first outbreak of ECF in naive cattle [7]. In Malawi, where the Southern region is considered non-endemic to ECF, it is not recommended to introduce animals that have been immunized with the MC vaccine as this may result in an introduction of the MC vaccine stocks which can be virulent in naïve animals. As the vaccinated animals become asymptomatic carriers, they can be a potential source of parasites in the areas where the vaccinated animals are introduced [1,7]. It is recommended that other control measures such as restriction of animal movement should also be taken together with the MC immunization.

Monitoring of the local genotypes of *T. parva* can help us to know if any of the local genotypes are undergoing expansion or not. Such information will provide valuable knowledge for the control of ECF. Monitoring the MC sporozoite live vaccine using molecular tools when deployed is important as it provides epidemiological data based on which effective control strategies of ECF in endemic areas can be made such as whether to continue with the MC approach or to use a local strain approach, as in the case of southern Zambia, where there were complications following the introduction of the MC vaccine [1]. Similarly, longitudinal studies, unlike cross-sectional studies, can provide more insight into the impact of the infection and treatment method (ITM) in the control of ECF in endemic countries, like the one done in Tanzania [35]. These studies can also help to address some concerns associated with the long-term use of the MC [35,36]. Thus, there is a need to carry out longitudinal studies in Malawi to have a complete profile and impact of the MC on the population genetics of *T. parva*. 

This study has also revealed that *T. parva* in Malawi is diverse due to the presence of both MC-related and non-related Tp1 and Tp2 sequences. Considering that the majority of the sequences generated in this study were MC-related, our findings may justify the continuous use of the MC in the sampled districts in the central region of Malawi, especially among the exotic and the crossbreed cattle. However, despite the observed endemic stability in the Malawi zebu cattle, especially the adult cattle, it may be necessary to immunise calves which easily succumb to ECF when infected by *T. parva* [13,18]. However, since the Tp1 and Tp2 antigens’ immune response is dependent on the phenotype of MHC class 1 haplotypes, further studies are required to determine these phenotypes in the Malawi zebu cattle population. Similarly, since the samples were only collected in a limited geographical area in central Malawi, these results may not fully reflect the genetic profile of *T. parva* in Malawi. Furthermore, since we identified new epitope variants whose impact cannot be theoretically predicted, it is recommended that a national survey be conducted with additional molecular tools that can help to provide more information on the *T. parva* genetic profile and the impact of the MC approach in the prevention of ECF in Malawi. Border districts, due to the illegal international livestock movement, and national parks with buffaloes are vital to determine the full genetic profile of *T. parva* in Malawi and the contribution of wildlife to the epidemiology of ECF in Malawi. Continuous monitoring of the use of the MC is a pre-requisite to the understanding of the parasite population flow in the country. 

## 4. Materials and Methods

### 4.1. Ethical Consideration

The clearance for animal sampling was obtained from the Ministry of Agriculture, Irrigation and Water Development (MoAIWD) in Malawi, through the Department of Animal Health and Livestock Development (DAHLD) reference number 10/15/32/D, with consent from the animals’ owners. 

### 4.2. Study Area and Sampling.

**Figure 6:**
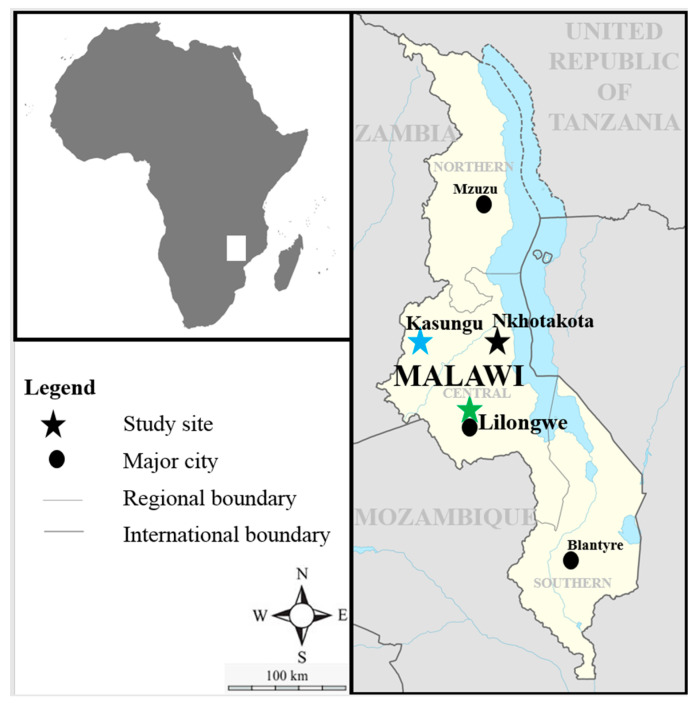
Map of Malawi showing districts where blood samples were collected.

Blood samples (n = 446) were collected from three districts in the central region of Malawi (Figure 6, Table 2) from February to March 2018 during the rainy season from apparently healthy animals. The samples (n = 199) were collected in the Kasungu district from the Chulu extension planning area (EPA) (12°49′3.912″ S, 33°18′10.008″ E) (n = 62), Lisasadzi EPA (13°16′20.682″ S, 33°8′11.446″ E) (n = 72) and Chipala EPA (13°7′2.761″ S, 33°19′7.314″ E) (n = 65). In the Nkhotakota district (n = 185), the samples were collected from the Mphonde EPA (12°48’19.8″ S, 34°11’27.2″ E) (n = 84) and Linga EPA (12°56′9.438″ S, 34°13′36.438″ E) (n = 101). With regards to the Lilongwe district (n = 62), all samples were obtained from Katete farm (14°01′23.8″ S, 33°45′17.3″ E), 30 of these were vaccinated with the MC 2 years and 3 months prior to the sampling period, while the remainder (n = 32) were unvaccinated but these animals have been co-grazing for more than 2 years. The animals in Kasungu and Nkhotakota were Malawi Zebu cattle that are managed under extensive grazing with animals from different herds, mix freely and had no ECF vaccination or dipping history to control ticks. The animals at Katete farm in Lilongwe were Holstein Friesian, that are kept under semi-intensive management with no contact with other herds. An acaricide application through dipping is used to control ticks weekly or fortnightly during the rainy season and during the dry season, respectively, and the MC is also used to control ECF in some animals. Approximately 5 ml of whole blood was collected by venipuncture of the external jugular vein after the disinfection of the puncture site with a methylated spirit cotton swab into an EDTA vacutainer tube. 

### 4.3. DNA Extraction.

DNA was extracted from 200 μL of whole blood using the Quick Gene DNA whole blood kit S (DB-S) (Kurabo Industries Ltd., Osaka, Japan) according to the manufacturer’s recommendations. The extracted DNA was stored at −20 °C until required for use.

### 4.4. PCR and Sequencing 

Molecular detection of *T. parva* was done by nested PCR assays targeting the *T. parva*-specific 104-kD antigen (p104) gene [37,38]. Primary PCR reactions were conducted in a 25 μL reaction mixture as described by Chatanga et al. [39], with minor modifications, where 1.0 μL was used as the DNA template. The annealing temperatures and expected product size are listed in Table 3. The cycling conditions for both the primary and secondary PCR were set with an initial denaturation at 94 °C for 1 min, followed by 35 cycles of denaturation at 98 °C for 10 sec, annealing for 15 sec, extension at 68 °C for 15 sec and a final extension at 68 °C for 5 min. The secondary PCR was conducted in a 10 μL reaction mixture containing 5.0 μL of 2×Gflex PCR Buffer (Mg2+, dNTP plus), 0.2 μL of Tks Gflex DNA Polymerase, 200 nM of each primer, 1.0 μL of 10-fold diluted primary PCR product which was used as the DNA template and water. The amplicons were electrophoresed in a 1.5% agarose gel stained with Gel-Red (Biotium, Hayward, CA. USA) and visualized under UV light.

PCR-positive samples were further used to amplify the two *T. parva* CD8^+^ antigen genes (Tp1 and Tp2) using newly designed semi-nested PCR assays. To improve the sensitivity of the assays, we designed two inner primers for semi-nested PCRs for both the Tp1 and Tp2 genes. The primer sequences used in this study are listed in Table 3. The primary PCRs were set as described by Pelle et al. [29], to amplify 432 bp and 525 bp of the Tp1 and Tp2 genes, respectively. All PCRs were conducted in a 10 μL reaction mixture as described above, with minor modification of the 1.0 μL DNA template. The cycling conditions for both primary PCRs were set as described by Chatanga et al. [39], with minor modifications to the annealing temperatures, as indicated in Table 3. The amplicons were electrophoresed in a 1.5% agarose gel stained with Gel-Red and visualized under UV light.

The amplicons purification was conducted as described by Mtsali et al. [40], while sequencing and sequence editing was conducted as described by Chatanga et al. [39]. The sequences generated in this study were submitted to the DNA Data Bank of Japan (DDBJ) [41] under the accession numbers LC522067 to LC522072 for Tp1 and LC522073 to LC522085 for Tp2. 

### 4.5. Data Analysis

Alignments of the consensus nucleotide sequences generated from the amplified DNA fragments were created using ClustalW in Molecular Evolutionary Genetics Analysis (MEGA v.7) [42], which was also used to translate the aligned nucleotide sequences into amino acid sequences. 

The genetic distances (expressed in terms of the number of differences per 100 bases or amino acids, including length polymorphisms) between every pair of sequences in a multiple alignment were generated using the DISTMAT program [43]. Estimates of DNA polymorphism, π, determined as the average number of nucleotide differences per site, were obtained with DnaSP v.6 [44]. The analysis of molecular variance (AMOVA) was performed using ‘Genalex6’ [45,46], in order to investigate the distribution of genetic variation among allelic sequences and to determine the level of population differentiation. To assess the similarity between the *T*. *parva* haplotypes found in Malawi with those of the MC strain’s maximum likelihood, a phylogenetic analysis and median-joining (MJ) network incorporating the Malawi Tp1 and Tp2 haplotypes and those from the components of the MC live vaccine strains, respectively, were constructed using MEGA v.7 [42] and NETWORK version 10.0.1.1 [47], respectively. Chi-square statistics were used to determine the correlation between the *T. parva* positive detection rate with the sampling site, vaccination status, age and sex of the animals. 

## 5. Conclusions

In conclusion, this study has provided molecular evidence that there is genetic diversity in *T. parva* in Malawi. It has also provided evidence that is in accordance with other studies, that there is a transmission of vaccine strains to unvaccinated cattle, as such a vaccination may be contributing to the genetic diversity of *T. parva* in Malawi. The finding of the sequences that are closely related to those isolated from buffaloes also emphasises the need to sample buffaloes to know the role that wildlife may play in the epidemiology of *T. parva* in Malawi. Thus, this study has provided information that can help in the control of ECF in Malawi and other endemic countries as well as those countries that are at risk of introduction of the disease. 

## Figures and Tables

**Table 1:** *T. parva* detection rate using p104 gene nested polymerase chain reactions (PCR) with regard to the host attributes.

Attribute	No. of Cattle	No. of *T. parva* Positive (%)	p-Value
**Origin**			0.000051*
Kasungu	199	128 (64.3%)	
Nkhotakota	185	78 (42.2%)	
Lilongwe	62	37 (59.7%)	
**Age**			0.000348*
Calves (<3 months)	12	3 (25.0%)	
Weaners (3 months–1 year)	83	32 (40.0%)	
Adults (>1years)	351	208 (59.3%)	
**Breed**			0.376194*
Holstein Friesian	62	37 (59.7%)	
Malawi zebu	384	206 (53.6%)	
**Sex**			0.543121*
Male	132	69 (52.3%)	
Female	314	174 (55.4%)	
**Vaccination status**			0. 037227*
Vaccinated	30	22 (73.3)	
Non-vaccinated	32	15 (46.9)	

* <0.05; Chi-square analysis determined the association, and p-values are shown.

**Table 2:** Tp2 cytotoxic T lymphocyte (CTL) epitope variants obtained in this study.

Epitope 1 (Tp2_27-37_) (4 Variants)	Epitope 2 (Tp2_40-48_) (3 Variants)	Epitope 3 (Tp2_49-59_) (3 Variants)	Epitope 4 (Tp2_96-104_) (2 Variants)	Epitope 5 (Tp2_98-106_) (2 Variants)	Epitope 6 (Tp2_138-147_) (3 Variants)
**SHEELKKLGML** (1,60,63,64,65,66)	**DGFDRDALF** (1,2,60,62,63,64)	**KSSHGMGKVGK** (1,2,60,62,63,64)	**FAQSLVCVL** (1,2,60,62,63,64,65,66)	**QSLVCVLMK** (1,2,60,62,63,64,65,66)	**KTSIPNPCKW** (1,2,60,62,64,65,66)
SDDELDTLGML (3,61)	PDLDKNRLF (3,61)	LTSHGMGRIGR (3,61)	FAASIKCVA (3,61)	ASIKCVAQY (3,61)	KPSVPNPCDW (3,61)
**SDEELNKLGML (2)**	*DGLNKDALF* (65,66)	*KTSKGMTEVGK* (65,66)			*KTSILNPCKW (63)*
*SREKLKKLGML (62)*					

Tp2 epitope variants detected in this study from amino acid alignment in Figure 2 above. Numbers in parenthesis after the epitope sequences correspond to amino acid variants carrying the epitopes (Figure 2). Epitope variants reported here for the first time are italicized, while the ones found in the MC vaccine stocks have been bolded.

**Table 3:** PCR primers used in this study.

Primer Name	Primer Sequence (5′ to 3′)	Target Gene/(PCR Type)	Product Size (bp)	Anneal. T. (°C)	Reference
**IL 3231**	ATTTAAGGAACCTGACGTGACTGC	*T. parva* p104 gene PCR	496	55	[37]
**IL 755**	TAAGATGCCGACTATTAATGACACC				
**IL 4234**	GGCCAAGGTCTCCTTCAGAATACG	*T. parva* p104 gene nested PCR	277	55	[38]
**IL 3232**	TGGGTGTGTTTCCTCGTCATCTGC				
**Tp1 F outer**	ATGGCCACTTCAATTGCATTTGCC	Tp1 gene PCR	432	50	[29]
**Tp1 R outer**	TAAATGAAATATTTATGAGCTTC				
**Tp2 F outer**	ATGAAATTGGCCGCCAGATTA	Tp2 gene PCR	525	50	[29]
**Tp2 R outer**	CTATGAAGTGCCGGAGGCTTC				
**Tp1 Inner F1**	CCGCKGATCCTGGATTCT	Tp1 gene semi-nested PCR	419	55	The outer primers, [29] Inner primers, this study.
**Tp1 R outer**	TAAATGAAATATTTATGAGCTTC				
**Tp1 Inner F2**	CATTTGCCGCKGATCCTG	Tp1 gene alternative semi-nested PCR	413	55	
**Tp1 R outer**	TAAATGAAATATTTATGAGCTTC				
**Tp2 Inner F**	CCGCCAGATTAATTAGCCTTT	Tp2 gene semi-nested PCR	511	57	
**Tp2 R outer**	CTATGAAGTGCCGGAGGCTTC				
**Tp2 F outer**	ATGAAATTGGCCGCCAGATTA	Tp2 gene alternative semi-nested PCR	505	57	
**Tp2 Inner R**	CCGGAGGCTTCTCCTTTTT				

**Abbreviations**: F: forward; R: Reverse; PCR: Polymerase chain reaction.

## Supplementary Materials

The following are available online at https://www.mdpi.com/2076-0817/9/5/334/s1, Figure S1: Multiple sequence alignment of Tp1 alleles obtained in this study from cattle from Malawi. The naming of the alleles follows the nomenclature by [27]. Alleles 1 and 5 were first described by [27]. The epitope coding region are boxed in red. The number in parenthesis is the number of sequences obtained in the allele., Figure S2: Multiple sequence alignment of Tp2 alleles obtained in this study from cattle from Malawi. The naming of the alleles follows the nomenclature by [27]. Alleles 1 and 2 and 3 were first described by [27]. The epitope coding region are boxed in red. The number in parenthesis is the number of sequences obtained in the allele.Table S1: Tp1 Analysis of molecular variation (AMOVA),Table S2: Tp2Analysis of molecular variation (AMOVA)Video S1: title.
