# Supplementary material for: Genetic Diversity and Sequence Polymorphism of Two Genes Encoding Theileria parva Antigens Recognized by CD8+ T Cells among Vaccinated and Unvaccinated Cattle in Malawi
Source: Pathogens. 2020 Apr 30;9(5):334. doi: 10.3390/pathogens9050334 (PMC7281522; doi:10.3390/pathogens9050334)
Supplement: Supplementary file 1 [file pathogens-09-00334-s001.pdf]

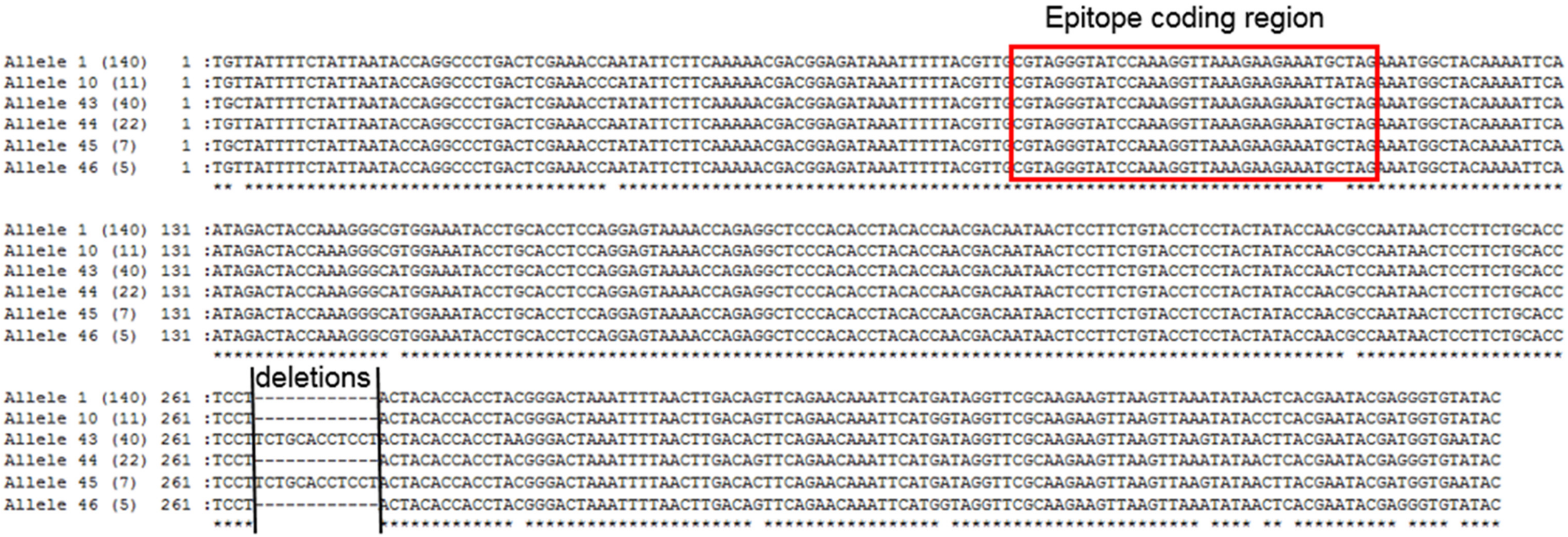

Supplementary Figure 1: Multiple sequence alignment of Tp1 alleles obtained in this study from cattle from Malawi. The naming of the alleles follows the nomenclature by [27]. Alleles 1 and 5 were first described by [27]. The epitope coding region are boxed in red. The number in parenthesis is the number of sequences obtained in the allele.

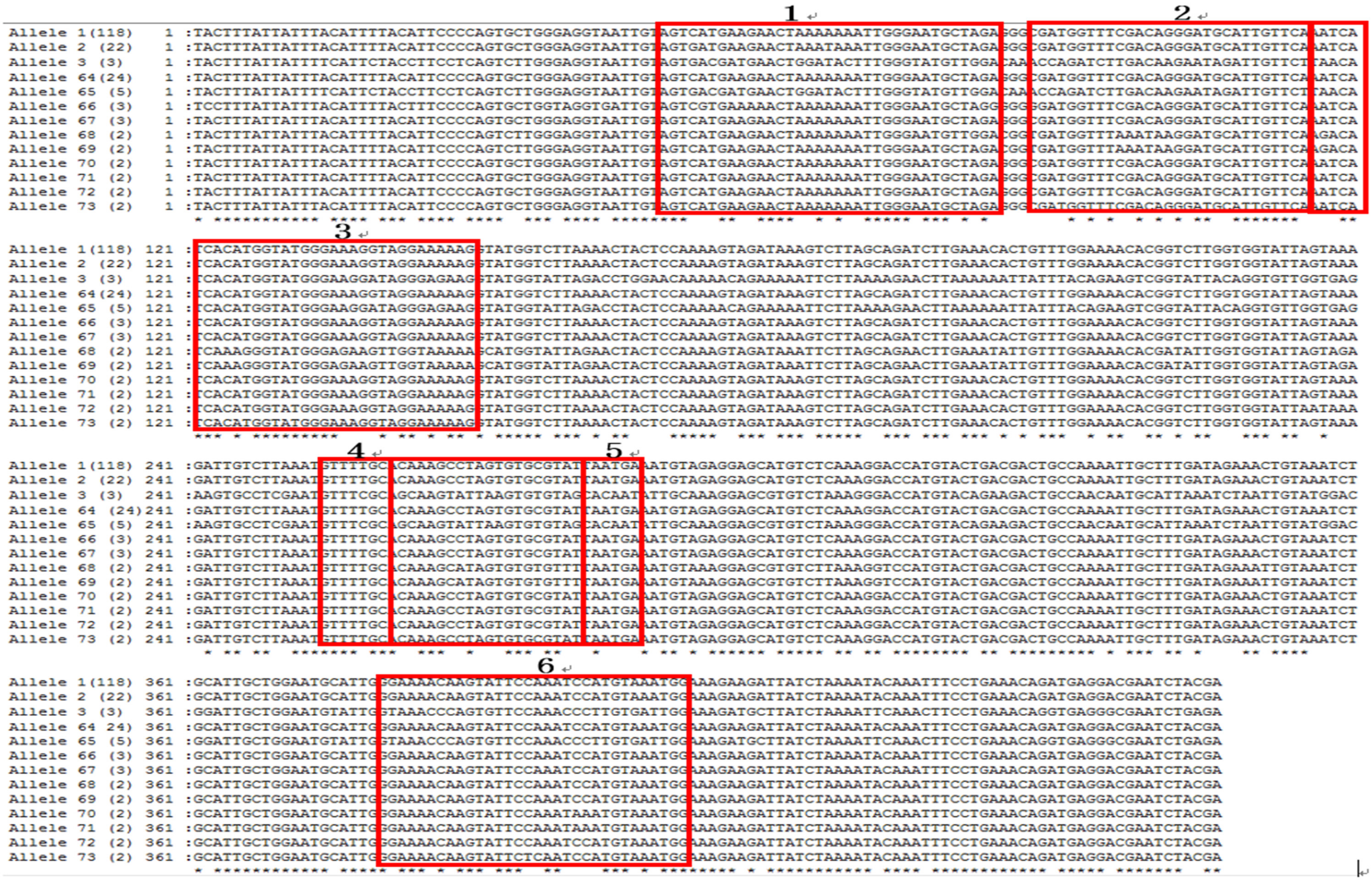

Supplementary Figure 2: Multiple sequence alignment of Tp2 alleles obtained in this study from cattle from Malawi. The naming of the alleles follows the nomenclature by [27]. Alleles 1 and 2 and 3 were first described by [27]. The epitope coding region are boxed in red. The number in parenthesis is the number of sequences obtained in the allele.

Supplementary Table 1: Tp1 Analysis of molecular variation (AMOVA)

**Source of variation**	**d.f**	**Sum of squares**	**Variance components**		**Percentage of variation**
**Among groups**	1	49.554	4.13637	Va	43.03
**Among populations within groups**	2	93.706	0.62209	Vb	6.47
**Within populations**	224	1087.612	4.85541	Vc	50.50
**Totals**	227	1230.873	9.61387		

Fixed indices

FSC: 0.11357

FST: 0.49496

FCT: 0.43025

Supplementary Table 2: Tp2Analysis of molecular variation (AMOVA)

Source of variation	d.f	Sum of squares	Variance components		Percentage of variation
**Among groups**	1	39.384	1.90318	Va	20.06
**Among populations within groups**	2	40.925	0.26387	Vb	2.78
**Within populations**	183	1339.381	7.31902	Vc	77.16
**Totals**	186	1419.690	9.48607		
Fixed indices					
**FSC: 0.03480**					
**FST: 0.22845**					
**FCT: 0.20063**					
